# To what extent does the COVID-19 pandemic impact patients with anorexia nervosa?

**DOI:** 10.1192/bjo.2021.725

**Published:** 2021-06-18

**Authors:** Krishna Mehta

**Affiliations:** Lancaster University

## Abstract

**Aims:**

This systematic review aims to discuss the extent to which the measures undertaken to control the COVID-19 pandemic in several countries have affected those with Anorexia Nervosa (AN). The coronavirus pandemic is still raging on in many countries and its effects will still be felt years from now and previous studies have shown that it has impacted other mental illnesses. AN cases are on the rise and the nature of the illness has deadly consequences, therefore it is paramount to discuss the relationship between the COVID-19 measures and symptomatology of AN to ensure appropriate services are in place to deal with potential outcomes.

**Method:**

Systematic search of the PubMed database gave thirty-three total results with seven of these used in this review. These studies met the inclusion criteria; examples include primary studies and use of the English Language. The exclusion criteria involved literature reviews, studies with less than ten participants and studies not separating AN from other eating disorders.

**Result:**

Many studies were cross sectional in nature except two longitudinal studies. Anorexic symptomology increased in the majority of papers in this review. Specifically restricting intake has increased compared to before lockdown measures. Physical activity has varied on an individual level in most studies potentially due to compensatory behaviours. Co-morbid psychopathologies were also noted during these studies. There are many factors behind these changes such as food insecurity, the effect of media and social media, uncertainty and the lack of social interaction. A number of participants across the studies were dissatisfied with treatment services during lockdowns. Variability in study results may be due to the differences in measures between countries and the use of self-reported cases compared to those contacted through care services. Limitations of this review are largely due to the how current the topic is and recommendations for the future include reviewing new studies that are published and to review the situation once the full impact of COVID-19 is known.

**Conclusion:**

Patients with AN have struggled more due to the COVID-19 measures and the true effect of the pandemic is yet to be felt. Further studies and reviews should be conducted. The appropriate services should be put into place to ensure patients are supported effectively.

